# Decoding overlapping memories in the medial temporal lobes using high-resolution fMRI

**DOI:** 10.1101/lm.023671.111

**Published:** 2011-12

**Authors:** Martin J. Chadwick, Demis Hassabis, Eleanor A. Maguire

**Affiliations:** 1 Wellcome Trust Centre for Neuroimaging, Institute of Neurology, University College London, London WC1N 3BG, United Kingdom; 2 Gatsby Computational Neuroscience Unit, University College London, London WC1N 3AR, United Kingdom

## Abstract

The hippocampus is proposed to process overlapping episodes as discrete memory traces, although direct evidence for this in human episodic memory is scarce. Using green-screen technology we created four highly overlapping movies of everyday events. Participants were scanned using high-resolution fMRI while recalling the movies. Multivariate pattern analysis revealed that the hippocampus supported distinct representations of each memory, while neighboring regions did not, demonstrating that the human hippocampus maintains unique pattern-separated memory traces even when memories are highly overlapping. The hippocampus also contained representations of spatial contexts that were shared across different memories, consistent with a specialized role in processing space.

Our daily lives usually involve encounters with a relatively limited range of people and locations, and consequently, the episodic memories that are formed often contain much overlap. Nevertheless, most of the time we are able to remember each event as a distinct episode. The hippocampus has long been implicated as the critical brain structure involved in separating overlapping episodes into unique representations, which are then stored as distinct memory traces (Marr 1971; O'Reilly and McClelland 1994; Treves and Rolls 1994; McClelland and Goddard 1996; O'Reilly and Rudy 2001). While the theoretical basis for this idea has a strong grounding in the anatomy of the hippocampus and in the rodent literature (Lee et al. 2004; Leutgeb et al. 2004, 2005, 2007; Vazdarjanova and Guzowski 2004; Wills et al. 2005), empirical evidence for the existence of traces of complex episodic memories in the human hippocampus remains scarce.

A recent study demonstrated that specific episodic-like memories can be decoded solely from patterns of functional MRI (fMRI) activity across voxels in the human hippocampus using multivariate pattern analysis (MVPA) (Chadwick et al. 2010), suggesting that episodic-like memory traces are present and detectable within the human hippocampus. However, each episode in this study differed along a variety of dimensions, including the identity of the people involved, the actions performed, and the spatial contexts. It was therefore not possible to determine exactly how the event-like episodes were represented within the hippocampus, or precisely what aspect of the episodes was being detected by the MVPA classification technique.

The purpose of the current study was to apply similar MVPA methods to the study of highly overlapping episodic-like memories in order to determine whether it is possible to detect unique, bound memory traces within the human hippocampus and elsewhere in the medial temporal lobes (MTL). The overlapping information in the episodes were a critical aspect of this study, as it was important to ensure that no episode could be uniquely specified by any single element within it. In order to create such fully controlled stimuli, we filmed two brief action events against a green-screen background. Each event was then superimposed onto the same two spatial contexts, creating four movie clips that included every combination of the two events and the two contexts (Fig. 1A). Each participant viewed the four movies prior to scanning, and then vividly recalled each one numerous times during high-resolution fMRI scanning. As the four episodes completely overlapped with one another in terms of their constituent elements, any successful differentiation of the four memories from patterns of activation must be due to the presence of unique, bound memory traces. If the hippocampus is exclusively involved in creating and maintaining distinct memory representations, it should be possible to decode highly overlapping episodic-like memories from the patterns of activity across voxels in the hippocampus, but not from other MTL regions.

**Figure 1.**
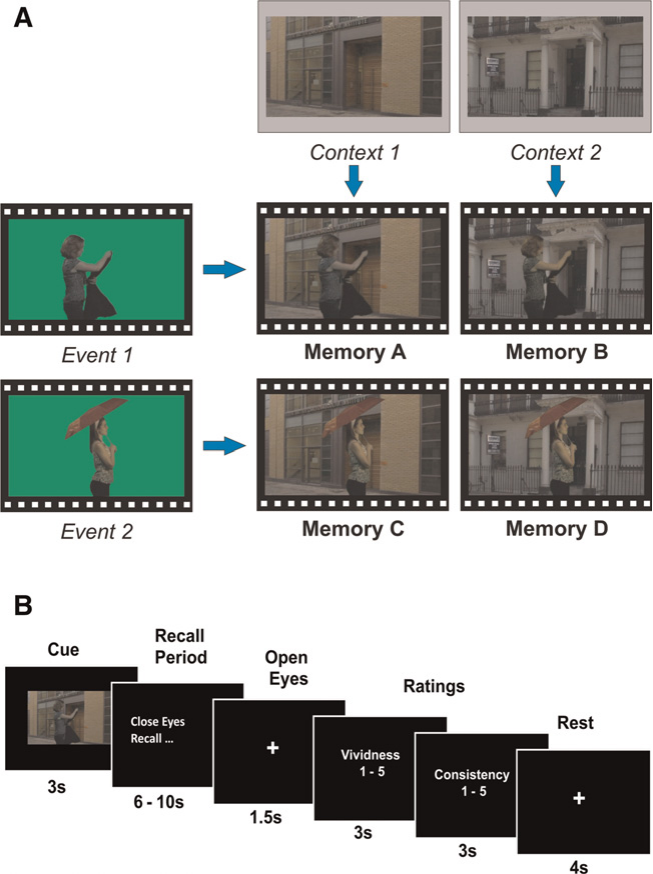
(*A*) The stimuli. Two events were filmed against a green-screen background (*left*). Each clip was 7-sec long and involved a short series of actions performed by a single female actress. In the first, a woman walked into the shot, removed her jacket, and placed it over her arm. In the second, a woman walked into the shot, took out and put up an umbrella. The two events were superimposed on two different spatial contexts (see contexts in *top-most* panels) in order to create four movies, which included all four combinations of event content and spatial context (see Memories A–D). These stimuli ensured that the memories would be dynamic and episodic-like in nature, while being fully controlled in terms of the event content and spatial context of each memory. (*B*) Timeline of a sample trial during fMRI scanning. On each trial, one of the four episodes was cued with a still photograph taken from the movie. Following this cue, participants were instructed to close their eyes and recall the episode as vividly and accurately as possible, after which behavioral ratings of the recall experience were taken (see Supplemental Material for details).

Fifteen healthy right-handed participants (eight female) took part in the experiment (mean age 21.17 years, SD 2.18 years, range 18–25 years). All had normal or corrected-to-normal vision and gave informed written consent to participation in accordance with the local research ethics committee. During a prescan training period, participants watched each of the four movie clips 12 times in total, and practiced vividly recalling a movie after each viewing. This degree of training was necessary in order to ensure that participants were able to recall every memory consistently and accurately on every trial. Participants then recalled the four memories repeatedly in a single session of high-resolution (1.5 mm^3^ isotropic voxels) fMRI scanning on a 3T MRI scanner (see Supplemental Material for details). Figure 1B shows the timeline of a sample trial during scanning. In total there were 20 trials of each memory, presented in a pseudo-random order, while ensuring that the same memory was not repeated twice or more in a row. This degree of repetition was necessary in order to provide enough training examples for the MVPA classifier.

T1-weighted structural MR images were manually segmented into four regions of interest (ROIs) within the MTL (Fig. 3A, below): hippocampus (HC), entorhinal cortex (EC), perirhinal cortex (PRC), and posterior parahippocampal cortex (PHC). All functional data were preprocessed using SPM8 (http://www.fil.ion.ucl.ac.uk/spm). The recall period of each individual trial was modeled as a separate regressor and convolved with the hemodynamic response function, creating participant-specific β estimates for each recall trial at each voxel (see Supplemental Material for details). These βs were converted to *t*-values (Misaki et al. 2010), thus creating a single *t*-value map for every accurately recalled memory trial during the functional session, and these data were used in the classification analyses.

We used a four-way linear support vector machine (SVM) classifier with leave-one-trial-out cross-validation to determine whether it was possible to discriminate between the four overlapping memories from the activity across voxels (Fig. 2A) in the four MTL regions (see Supplemental Material for more information, for details of other control analyses examining the effect of smoothing, and of the number of voxels in the different regions of interest). We found a significant level of decoding in the hippocampus (*t* = 1.90; *P* = 0.04), which was in contrast to the other MTL regions, none of which supported significant levels of decoding (EC: *t* = −1.09, *P* = 0.85; PRC: *t* = 1.55, *P* = 0.07; PHC: *t* = 0.31, *P* = 0.38) (Fig. 3B). This result shows that in this extreme example of overlapping memories, where no single element allowed the differentiation of a memory, the hippocampus contained distinct representations of each individual memory. Therefore, this demonstrates that unique, bound memory traces of complex, realistic event-like episodes are present and detectable within the human hippocampus.

**Figure 2.**
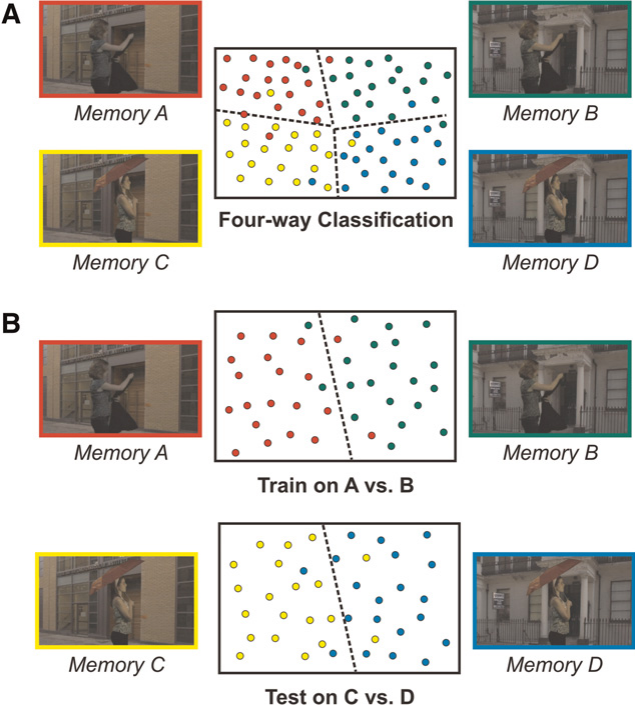
An overview of the multivariate analyses. (*A*) An illustration of the four-way classification procedure. The classifier was trained to find patterns of activated voxels for each of the four memories that best differentiated it from the other three memories. In this simplified schematic, each of the four memories is color coded, and each colored dot represents the activity profile of a single recall trial projected into multidimensional space. The classifier was trained to find divisions within this space that best differentiated the activity patterns associated with each memory, here represented by the dotted lines. In this case, each of these four regions is dominated by activity related to one of the four memories, demonstrating that the classifier has been able to find distinct patterns for each individual memory. (*B*) An illustration of the spatial context classification procedure. In this analysis we were interested in seeking information about spatial context that was common across different memories. In order to do this, a classifier was trained to differentiate Memories A and B, where the event content is exactly matched, and the memories only differ in terms of spatial context. If any spatial context information is present across pairs of memories, then the classifier that has been trained on A vs. B should successfully classify Memories C vs. D, as the spatial contexts are exactly the same—i.e., A and C share Context 1, and B and D share Context 2.

**Figure 3.**
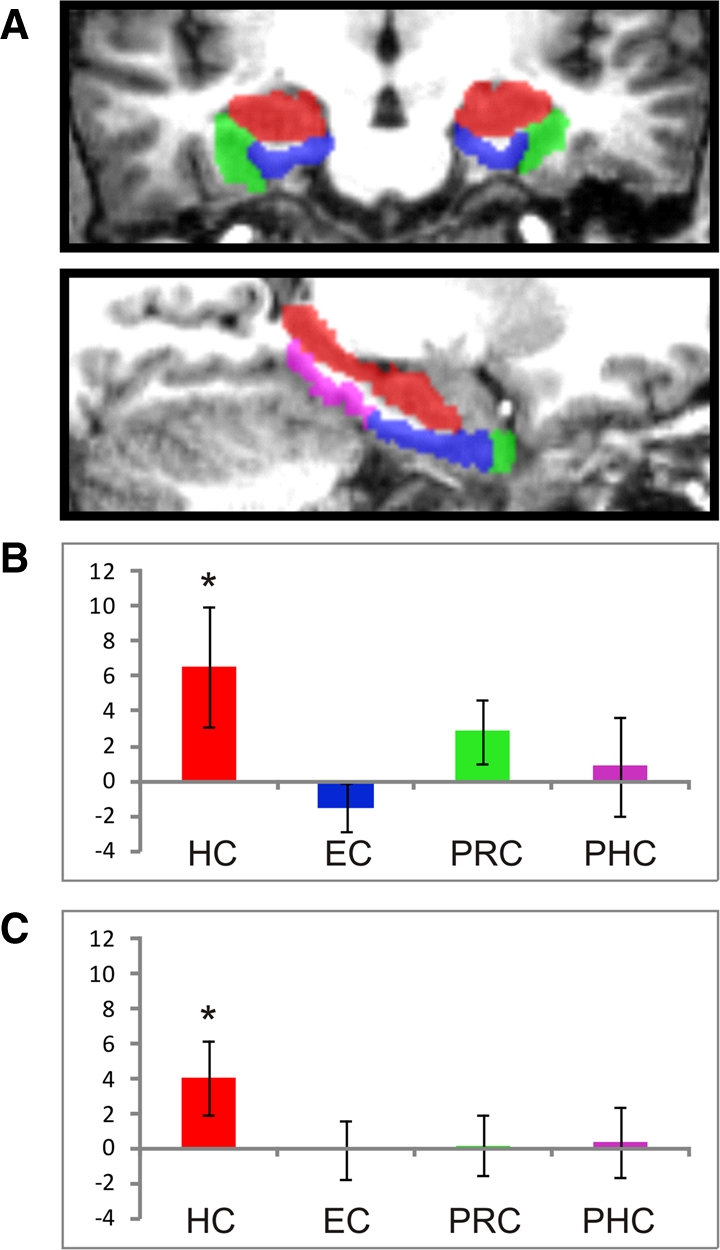
Summary of results. (*A*) Segmented regions of interest in the medial temporal lobe of one of the participants shown in the coronal plane (*top*) and sagittally (*bottom*). The hippocampus (HC) is shown in red, entorhinal cortex (EC) in blue, perirhinal cortex (PRC) in green, and the parahippocampal cortex (PHC) in magenta. Group mean decoding results for each of the four MTL regions are displayed for the four-way classification analysis (*B*) and the spatial context classification analysis (*C*). Results are displayed as percentage above chance accuracy, with standard error bars. In both analyses, only the HC results are significantly above chance. Note that for both significant results, significance tests were repeated using a nonparametric permutation approach (see Supplemental Material), and in each case the results remained significant.

The fact that distinct representations of all four highly overlapping episodes are present within the hippocampus provides support for an influential computational account of hippocampal function (Marr 1971; O'Reilly and McClelland 1994; Treves and Rolls 1994; McClelland and Goddard 1996; O'Reilly and Rudy 2001). This theory proposes that overlapping events are orthogonalized into distinct representations within the hippocampus by a process known as pattern separation. According to this view, each episodic memory is coded by a unique representation within the hippocampus that is distinct from the individual elements making up that episode. Results from both electrophysiological recordings and immediate early gene imaging analyses have demonstrated that the rat hippocampus displays pattern separation in a variety of spatial tasks (Lee et al. 2004; Leutgeb et al. 2004, 2005, 2007; Vazdarjanova and Guzowski 2004; Wills et al. 2005). A small number of fMRI studies in humans have produced results that are consistent with similar processes occurring in the human hippocampus (Kumaran and Maguire 2006; Bakker et al. 2008; Bonnici et al. 2011; Lacy et al. 2011), all of which involved graded changes in pictures of objects or scenes. However, none of these studies could speak to the issue of whether pattern separation operates in the context of more complex dynamic episodic-like memories, a link that is critical to computational theories of hippocampal function. The current results demonstrate that the hippocampus is able to maintain distinct episodic-like representations despite the high degree of overlap between the four episodes, in accord with computational theories.

An important aspect of the study design was that it enabled us to make further inferences about the representations within the hippocampus. In addition to its role in episodic memory, it has long been known that the hippocampus is critical for spatial representations and spatial navigation (e.g., O'Keefe and Dostrovsky 1971; O'Keefe and Nadel 1978; Burgess et al. 2002; Hassabis et al. 2007, 2009). In a previous (standard, univariate) fMRI study, the hippocampus was more active during recognition memory for both episodic and semantic information that included a spatial context compared with memories that did not explicitly require consideration of the spatial context (Hoscheidt et al. 2010). In that study, however, the contexts and content were not completely controlled, and it is not clear whether the spatial context drove the hippocampal activations or an interaction between context and content. In contrast, the memories in our experiment were completely controlled in terms of spatial context and event content. Given this, and the multivariate approach to data analysis, we were able to ask a more challenging question—as every memory shared its spatial context with one other memory, was there evidence of a common spatial context representation across such pairs of memories in the MTL?

In order to test this, a classifier was trained to differentiate Memories A and B, where the event content is exactly matched, and the memories only differ in terms of spatial context (Fig. 2B). If any spatial context information is present across pairs of memories, then the classifier that has been trained on A vs. B should successfully classify Memories C vs. D, as the spatial contexts are exactly the same, i.e., A and C share Context 1, and B and D share Context 2. Only the classifier operating on the hippocampal voxels displayed successful decoding of the common spatial representation (*t* = 2.39; *P* = 0.02) (Fig. 3C), with no significant decoding in the other MTL regions (EC: *t* = −0.05, *P* = 0.52; PRC: *t* = 0.16, *P* = 0.88; PHC: *t* = 0.21, *P* = 0.42). This demonstrates that during episodic recall, in addition to representing the four individual memories, the hippocampus also contains a general representation of spatial context that is active during the recall of any memory sharing that spatial context. While this result is consistent with a wealth of evidence suggesting that the hippocampus is critical for spatial representations, as far as we are aware no previous study has isolated the representation of purely spatial information in this way during episodic recall. This provides a novel insight into spatial processing, and demonstrates that even during recall of internally generated, complex episodic-like memories, the hippocampus maintains a distinct representation of relevant spatial environments. It is interesting to note that we did not find any evidence for the presence of generalized spatial information within the parahippocampal cortex, a region documented to represent scene information in previous MVPA studies (e.g., Hassabis et al. 2009; Bonnici et al. 2011). One clear difference between this study and those previous studies is that here we examined spatial scenes that were generated as part of episodic-like memories. It is possible that spatial representations generated during episodic recall are more strongly represented within the hippocampus than in neighboring regions. This will require elucidation in future studies.

We also conducted a similar analysis to look for representations of common event content information, by training on memories A and C (where spatial context is exactly matched and the memories only differ in terms of event content) and testing on memories B and D, with no significant results in any of the four MTL regions (HC: *t* = 0.51, *P* = 0.31; EC: *t* = 0.95, *P* = 0.18; PRC: *t* = 0.69, *P* = 0.25; PHC: *t* = 1.59, *P* = 0.07). This suggests that while information relating to the fully bound memories and also the spatial contexts is relatively high and decodable in the hippocampus, information relating to event content alone is less so, at least in the four MTL regions that we examined. This does not preclude such information existing elsewhere, beyond our partial volume. Moreover, it is possible that had we been able to look within hippocampal subfields, we might have detected evidence for content information, in line with previous studies such as Bakker et al. (2008). Notably, this failure to decode the event content from hippocampal activation bolsters the argument that the successful spatial context decoding analysis was indeed driven by the spatial properties of the context rather than any individual objects from the background (e.g., doors, railings). Each event also contained distinctly different objects (e.g., umbrella, jacket), and yet it was not possible to decode these events, suggesting that any signal regarding individual objects present within the hippocampus was not sufficient to drive successful classification performance.

Given that the spatial contexts common to different memories were represented in the hippocampus, this raises an important issue regarding the initial analysis where all four memories were decoded using a four-way classifier. Theoretically, it would be possible to get above-chance decoding accuracy in the four-way analysis purely on the basis of spatial information, rather than specific information about each of the four memories, as four-way SVMs are based on a series of two-way classifications (Hsu and Lin 2002). In order to rule out this explanation, we investigated the patterns of misclassification in the four-way analysis. On each trial, the classifier can either correctly classify the memory, or it can misclassify it. Misclassifications can be one of three types: (1) incorrectly classified as a memory that shares the same spatial context (spatial misclassification); (2) incorrectly classified as a memory that shares the same event content (content misclassification); or (3) incorrectly classified as a memory that shares neither spatial context nor event content (orthogonal misclassification). If the four-way classification results are being driven by spatial information, then one would expect the misclassifications to be biased toward memories that share the same spatial context, and there should be a greater proportion of spatial misclassifications than either content or orthogonal misclassifications. However, the proportion of spatial misclassifications was not significantly different to either the content or orthogonal misclassifications (spatial > event misclassifications, *t* = −1.462, *P* = 0.92; spatial > orthogonal misclassifications, *t* = −2.754, *P* = 0.99). This demonstrates that the results of the four-way analysis were not driven by the representation of common spatial information, but, instead, genuinely reflect the representation of four distinct episodic memories within the hippocampus.

One key question regarding these results is to what extent these representations reflect true episodic memories. Episodic memory is commonly defined as the memory for personally experienced events, including details of the event along with the concomitant spatial and temporal context (Tulving 2001). The events used in this study are not truly “episodic” under this definition, because each movie clip was presented 12 times during prescan training (to ensure that the memory representations were stable, necessary for MVPA—see Supplemental Material), while genuine episodes are experienced only once. Nevertheless, we ensured that only those trials where there was vivid recall of the original movies were included in the analyses. We believe that the core processes involved in the vivid recall of these episodic-like memories overlap considerably with those involved in episodic recall.

In summary, our findings demonstrate that unique, bound episodic-like memory traces are present and detectable in the human hippocampus. This provides empirical evidence of a link between complex episodic-like memories and pattern separation in the human hippocampus, and offers clear support for computational theories of hippocampal function (Marr 1971; O'Reilly and McClelland 1994; Treves and Rolls 1994; McClelland and Goddard 1996; O'Reilly and Rudy 2001). Moreover, the results re-emphasize the fundamental role of the hippocampus in representing space (O'Keefe and Dostrovsky 1971; O'Keefe and Nadel 1978; Burgess et al. 2002; Hassabis and Maguire 2007; Hassabis et al. 2009), and demonstrate that spatial contexts are represented within the hippocampus during episodic recall. Together, this set of findings suggests that the hippocampus is capable of supporting at least two different types of representation—each memory has a unique representation created through a process of pattern separation, and at the same time spatial backdrops that are common to different memories are also represented in the hippocampus.

While the current results provide novel insights into the representational content of the hippocampus as a whole, important questions remain. Within the nonhuman hippocampus, region CA3 in particular has been implicated in the formation and maintenance of distinct, pattern-separated representations, while CA1 is thought to contain less-distinct representations (Leutgeb et al. 2004; Vazdarjanova and Guzowski 2004). It is possible that in humans also, different hippocampal subfields may play dissociable roles in maintaining distinct episodic memories on the one hand and spatial representations on the other. In the future it will be important to address these questions to build a complete picture of the underlying representational and computational properties of the hippocampal subfields.
